# Prevention and Treatment of Decay on Proximal Surfaces of the Teeth

**Published:** 1875-07

**Authors:** G. H. Cushing


AMERICAN JOURNAL

OF

DENTAL SCIENCE.

Vol. IX. THIRD SERIES—JULY, 1875. No. 3.

### ARTICLE I.

### *Prevention and Treatment of Decay on Proximal Surfaces of the Teeth.*

BY G. H. CUSHING, D. D. S.

The subject is divided into two distinct branches. The first looks only to prophylactic measures—*i. e.*, to the means of prevention. For the proper understanding of this, it is essential that we should define what is, in this paper, understood to be the etiology of dental caries. Without discussing the various theories which have been advanced from time to time, it will be assumed that, although the immediate process of so-called caries is unquestionably a chemical one—being the reaction of an acid upon the lime salts of the tooth—yet that such reaction would not take place, were it not for some constitutional abnormality which impairs the vital force to the extent of weakening the power of resistance to destructive agents, which is inherent in the teeth. Taking, in connection with this, the imperfect constitution of the teeth as a predisposing cause, and I think we have the fundamental elements of the etiology of dental caries. In support of this assumption, we must consider man in his original, primitive condition, when his organization was supposed to be perfect. I take it for granted that the following proposition must be universally accepted as correct, viz: that there was a time when the animal part of man was perfect in form, development and function, and that when living out such a life as would seem to have been intended for him, his teeth would, at its close, present no indications even of what we now know as dental caries. I assume as equally incontestible the position that his teeth, during all his life, were constantly subjected to contact with acids of various kinds—those of fruits, which then, as now, in certain localities, formed the principal part of his diet—and to those produced in the mouth by the processes of fermentation and decomposition, which processes must take place in the mouths of those even in perfect health.

These premises being granted as sound, force the conclusion upon us that the teeth have, in the natural order of things, the power inherent in them of a certain resistance to destructive agents.

Now it is by no means certain that the teeth of men of the present day are subjected to contact with acids to any greater extent than were those of the original man, or that these acids are more powerful in their destructive agency than were those of that earlier time, and yet decay of the teeth is the universal rule at the present day, and probably co-extensive, throughout the world, with the history of civilization. These propositions being thus far granted, we have established the position that the present generation are characterized by an almost universal condition of weakened vital force, as evinced in the proneness of the teeth to succumb to the attacks of agents which, under fully healthy conditions of the body, ought to have no effect upon them at all. The part which imperfect constitutional structure plays in the matter, I do not regard as of great importance; and this condition is chiefly to be noticed as favoring a more rapid destruction of the teeth, where caries once commences. I think the fact of imperfect organization of the teeth, which is often apparent, furnishes a strong support to the first assumption "that caries is mainly dependent upon constitutional causes, which tend to impair the power of resistance of the teeth to destructive agents;" because we often see in the mouths of certain individuals teeth which are evidently of imperfect character, being less dense in structure and showing imperfect fusion of the enamel at the fissures and depressions, which yet remain undecayed, or decay only to a slight extent, although subjected, so far as we can ascertain, to the same destructive agents, which rapidly disintegrate teeth of even better quality in the mouths of other persons.

These phenomena can only be accounted for upon the theory that there is in one case a resistant force at work independent of local conditions, which is absent or held in abeyance in the other. One other fact may be cited, as tending to support the theory here advanced—and that is, the greater tendency to, and more rapid progress of, caries at certain times in the same mouth, and even in the same teeth, as particularly noticed in the case of pregnant women.

Now, it will very properly be asked, "What is the *cause* of this constitutional disturbance, which thus weakens the power of resistance in the teeth?"

The only reply which would seem to be possible to this query, in the present condition of our knowledge is, impaired nutrition.

Vague and indefinite as such a reply must be, it yet probably conveys as intelligible an idea as any more elaborate one would do, and probably as nearly expresses the truth. Back of this, again, comes the inquiry, why this perverted nutrition? To this we can only answer, because of disobedience of nature's laws, in almost every particular. I need not elaborate this point. Every man is conscious that not an hour of his life passes, in which he does not violate some law of nature, setting aside constantly the simplest teachings of common sense for the gratification of appetite, or as a matter of convenience, or from the influence of long continued habit, or from indolence.

To this violation of "Divine law" are to be traced "all the ills that flesh is heir to."

This brings us to the starting point of our subject, that is the consideration of the first means of prevention of decay. It might appear at the first glance, if we accept the foregoing premises as sound, that the prevention of disease would be easy of accomplishment, provided we could induce men to become obedient to law, and to submit themselves to the teachings of hygienic science. The extent of improved bodily and mental condition, which such a change in the order of things would secure, can never be estimated. It would of course be immense, but even were it possible to induce such a change, we should find the benefit to the teeth for a long time after such a revolution was effected, to be comparatively slight, for the effects of inherited disease and increasing degeneracy, extending through many generations, could not be overcome at once, and while the general condition of the body might be made to more nearly approximate health, yet the teeth would be found still to be imperfect, and to be continually yielding to destructive influences.

Theoretically, this treatment should secure the end sought, but practically it would not. Now we must not look at this subject from a purely theoretical stand-point. One of the most important things for us to bear in mind, in attempting to understand subjects connected with the prevention and healing of disease, is that we must recognize the fact that we have to take things as we find them, and to deal with them accordingly. We know that men will continue to act contrary to the teachings of reason, though assured that the penalty must be paid; that however fully we may succeed in convincing their reason we shall fail to control their actions to any great extent, and yet that they will expect us to afford them relief from suffering, whether induced by their own acts or otherwise. This relief we hould be prepared to give to the fullest possible degrees.

So, while we must accord to hygiene all that is due, and must use our utmost endeavors to try to lead men into accord with its teachings, we must not ignore the self-evident truth that our efforts in this direction can be of but slight avail. In view of these facts, we are compelled to seek relief from other sources.

The question then presents itself, what means have we other than those already considered through which to secure prophylaxis ? It has been affirmed thot the principal cause of dental caries is to be found in a lessened power of resistance to destructive agents, due to constitutional disturbance arising from perverted nutrition.

Can we then, after having secured on the part of our patients the fullest possible compliance with hygienic laws, and having failed to secure the results to be desired, hope for further benefit from constitutional treatment.

It has long been my belief that the time would come when it would be possible to secure partial relief at least by means of such treatment. We must remember that very little comparatively, can be hoped for from our teaching of hygienic laws, and if it is possible for us to supply-nature's deficiencies or counteract the influence of disease by means of medication, or by the administration of concentrated or artificial food, we should leave no effort untried to establish a practice in this direction, which may be relied upon as efficacious and safe in a majority of cases.

It is somewhat surprising, and much to be deplored, that no more has been attempted in this direction by men who are qualified for such experimentation, for it is unreasonable to suppose that with the advanced knowledge of the present day to guide us, and with the resources of chemistry at our hand, no means could be found which would aid us in our endeavors to anticipate disease. It is true that the use of the phosphate of lime has been advocated to some extent, as a prophylactic. Dr. Wescott and Dr. Watt, I believe, have both advocated its use, and claimed that its administration at their hands resulted in marked benefit to their patients.

But the generally accepted views of practitioners of the present day, both of dentistry and general medicine, deny the value of the treatment as a general rule, where the crude phosphate of lime is administered, as it is claimed that it is not taken up by the circulation, that it is insoluble, and is carried off in the excretions unchanged. An article in the *Missouri Dental Journal* for November, 1872, from the pen of Dr. R. Blacke, Laureate of the Medical School of Paris, seems to hold out promise of beneficial results from the use of the recent preparation of the syrup of the lacto-phosphate of lime. The article seems encouraging to us who seek for aid in this direction. The reasoning of the author certainly appears convincing, and his argument does not rest upon theory alone, but is based upon experiments which seem fully to warrant what he claims for the remedy. It is very possible that in this remedy we may find one of the agents which we seek, as a means for the prevention of dental disease. It is certainly worth our while to give it a thorough trial, for without empirical knowledge of its operation, we can form no correct judgment upon its merits. Meanwhile, our attention should be earnestly directed toward the investigation of the various abnormal conditions which unquestionably influence, in some way or other, the progress of dental caries, and our endeavor should certainly be to arrive at a knowledge of such remedies as will cure, or at least ameliorate those conditions.

In what I have said of constitutional treatment, I am aware that I have advanced little that is of any value, except in so far as my remarks may help to stimulate thought and action in this direction, on the part of many men in the profession who are qualified to prosecute such investigations and experiments as shall eventually bring us the desired aid.

It is with the hope that such may be their effect, that I leave the consideration of this point, and turn to consider what other means, if any, there are that are available as in any sense preventive.

It has been established as a fact, from long continued observation, that the teeth are especially liable to deeay, in a large majority of cases, upon their proximal surfaces, where those surfaces are in contact. It has also been observed, that where there exists a free and ample space between the teeth, those surfaces are as a rule exempt from caries. These are established facts, not mere theories. These conditions are observed in mouths of every variety of character, and with teeth of corresponding variety. Where the secretions are vitiated and unduly acid, and as well where they are apparently normal and neutral. So fully recognized are these facts at the present time, that it is doubtful if any person could be found to-day who would deny the proposition, that if free spaces naturally existed between the teeth, there would be comparatively little danger of caries attacking those surfaces.

These facts would justify, as entirely reasonable, the proposition that if such spaces could be artificially secured, without injury to the teeth by reason of the operations necessary to that end, then we might feel confident that we had secured a method of prevention which would greatly lessen the sum of human misery, and add largely to the beneficient influences of our calling. In view of the fact that it is more difficult to arrest decay upon the surfaces now under consideration, by the process of filling, that when it occurs in any other locality, and in view of the fact, too, that the majority of teeth which are eventually lost under the hands of skillful practitioners are lost in consequence of decay upon these surfaces, it becomes us most earnestly to inquire if any such method of separation for prevention may be safely pursued, and what grounds there may be to support the hope that such a practice would prove successful.

To secure such permanent artificial spaces, resort must be had to the cutting away of the teeth, by the use of the file or other instruments. The use of the file for this purpose is of old date in the history of the profession, though its application in times past was confined chiefly to the incisor teeth. All the text-books refer to the practice (more generally, however, for the removal of superficial decay, than as a means of prevention,) and none of these entirely condemn it.

Undoubtedly the use of the file, or its equivalent, has been more or less resorted to for the purpose of prevention of decay, by means of creating spaces between the teeth, from the commencement of our life as a profession to the present day ; but it has never been systematically recommended until quite recently, when Dr. Robert Arthur, of Baltimore, promulgated to the profession and the world, his "*system* for the prevention and treatment of decay upon the proximal surfaces of the teeth." His method contemplates the general separation of the teeth, by means of cutting away their approximating sides.

In determining the merits of his claims, it is necessary first to satisfy ourselves whether the teeth may be cut away, as he proposes, with safety, or without injury. There was, for a long time, a very general prejudice against the removal of the enamel of the tooth, because it was thought that the dentine thereby exposed, would more readily decay—in fact, that it would be quite certain to do so in many cases, simply by reason of the removal of the enamel. Such a prejudice still exists to some extent, though probably to a very limited degree, compared with a period a few years previous to this.

The fact would seem to be not established, by reason of observations covering a period of many years, that a properly polished surface of dentine, especially when not in contact with any other surface, will not perceptibly be attacked by caries sooner than a surface of enamel under similar conditions. This I assume to be true. Granting this, we must acknowledge that there can be no objection to the cutting or filing the teeth for the reason of supposed injury thereby resulting. Having then satisfied ourselves that there is no just ground of opposition to the *means*

which Dr. Arthur proposes to employ, let us further examine *how* he proposes to apply them, and what he expects to accomplish thereby.

Briefly stated, his expectation from this treatment may be summed up thus, quoting mainly his own language: "Decay of the teeth may be *prevented* from occurring at places where it is most destructive, and requires the most difficult, painful and expensive operations for its arrest;" *i. e.*, the proximal surfaces. This is the chief claim, and the one mainly interesting us at this time. I cannot suppose that any well grounded opposition to this expectation can be sustained, provided ample, *permanent* spaces can be secured. It has been already affirmed that ample spaces of a *permanent* character between the teeth, of natural occurrence, would undoubtedly, as a general rule, prevent the occurrence of caries on the surfaces now under consideration. This is again reaffirmed as being a proposition, in view of what experience teaches, of a self-evident character.

His method of *application* of these means requires a somewhat more extended and careful consideration. Let us commence with him at the very first step. He proposes, upon the appearance of the first permanent molars, to cut away the temporary molars, which would then be in contact with them, creating so ample space as to insure the mesial surfaces of the first permanent molars from decay, certainly up to the time of the eruption of the second bicuspids.

There can be no objection urged against this treatment, certainly. But I cannot do better than to quote Dr. Arthur's own language upon this point. He says: "If decay should never attack the permanent molar teeth at the points referred to, even if the separations were not made, the cutting away of these temporary teeth cannot be in any way injurious, as the enamel of the permanent teeth is left intact. * * * But, as almost universally happens, and as has been shown, the permanent molar teeth do decay frequently and rapidly at the points referred to, the service advised must be admitted to be invaluable, if they are protected from an attack of decay."

This seems to me so entirely reasonable, that I conceive it to be only necessary to secure your thought upon this point for a moment, to gain your approval of his system thus far.

The next step in his method is to treat the temporary laterals, when they are in contact with the permanent centrals, as the temporary molars were treated—*i. e.*, to cut them away freely, thereby securing safety to the distal surfaces of the permanent centrals, until the permanent laterals take their place in the arch. This can have no objection urged against it, any more than in the case of the temporary molars. The next step takes us to the mesial surfaces of the permanent central incisors, which are in contact, and which he proposes to treat in the same manner, so cutting them away on their lingual aspects as not to disfigure their appearance in the slightest degree, and yet to leave them in contact at the least possible point; while the proximal surfaces shall be, to a great extent, self-cleansing, and always easy of examination. To this practice, if the premises which, it is claimed, have heretofore been established, are correct, there can certainly be no well grounded objection.

I think it can be safely affirmed—and I do not anticipate that a denial will be offered by any in the profession throughout the land—that such spaces, so secured, and the cut surfaces properly polished and re-polished as often as may become necessary, will render these teeth almost absolutely secure from decay upon their proximal sides. The treatment follows on with the eruption of the permanent teeth, and repeats itself with the six anterior teeth; and no objections can be urged against these subsequent operations that could not have been urged against the very first operations upon the permanent incisors.

We now come to the treatment of the bicuspids and molars, which he proposes to treat upon the same principle.

The only point claiming our particular attention with regard to these teeth is the shape which he gives to the spaces. It is designated as the double V, or double wedged-shaped—that is, as though two wedges had been cut from between the teeth, the base or broad end of one of which should lie toward the palatine or lingual aspect, and the broad end of the other wedge at the grinding surfaces of the teeth. The spaces are shaped thus for the purpose of securing self-cleansing surfaces—or, in other words, so that the food which, in mastication, must necessarily be forced into these spaces, will still be inclined to be easily forced out of them, and so rather be pushed over the surfaces, and not be permanently lodged and retained, and also so that the secretions of the mouth, and more comminuted particles of food remaining after eating, may, by the action of the tongue and the flowing of the saliva, be easily removed, and consequently less of such material be retained to become destructive through the processes of decomposition constantly going on in the mouth. That this peculiar shape given to these spaces is the best that has yet been devised, I think there can be no doubt. It seems to be entirely in accordance with well-known principles of philosophy, and until some other method of shaping such spaces shall be demonstrated to be better, these must remain as unquestionably the correct shapes, when it is decided to separate the teeth at all. Here I must quote from Dr. Arthur, who says, in speaking of the points essential to success: "The separations must be thorough. No obstructions to the free passage of a piece of thread, or linen, or the tooth-pick, must be permitted to remain. These spaces must be made free *from the grinding surfaces to the gums.* * * * * The teeth may be permitted to touch at some point, but the contact must be so slight as readily to allow the passage at pleasure of the substances employed to cleanse them. The surfaces of the separated teeth must, if possible, be rendered plain. *It is essential that they should be thoroughly polished.*"

I will here quote again from the same author, from different parts of his book. On page 103 he says: "Now let it be clearly understood that this separation of the incisor teeth is *not advised* indiscriminately. The incisor teeth of every child do not necessarily or inevitably decay. There is a small, but it is a very small, number of individuals, who, even at the present day, when the prevalence of decay is so common, pass through life without being subject to decay of the teeth at all; and sometimes, though more rarely, the incisor teeth remain sound during life, while the back teeth are destroyed by decay, commencing on the surfaces in contact. A dentist who is worthy of the name should be able to recognize such cases, and to avoid unnecessary and injurious intermeddling with any of the teeth."

On page 154 he says again: "It will be seen that I do not advise the indiscriminate and almost unlimited filing of the teeth, as has been supposed." And on page 169 he sums up thus: "What I propose is, *the separation of teeth closely in contact*, which are of such frail character, and are exposed to such destructive influences, that their decay is inevitable, or has already occurred."

I have now briefly and imperfectly presented the main features of Dr. Arthur's method, and have endeavored to fairly consider the chief and obvious objections to the proposed operations, as they have been followed in their order. It now remains for us to consider what objections can be urged against the system as a whole, and to discover what lessons experience teaches, by which we may form some reasonable conclusions, regarding the hopes which may be entertained concerning such practice.

Among those in the profession who fully agree with Dr. Arthur's method, as applied to the incisor teeth, there are some to be found who make the objection (inquiringly) as regards the application of the method to the bicuspids and molars, that the spaces he proposes to make will not remain permanently, but will sooner or later become closed, or so nearly so as to defeat the ends for which they were made.

Others again dogmatically assert that they will so close, and no good can result of a permanent character from this treatment of the bicuspids and molars. Now let us consider for a moment what would *seem* to be the probabilities in the case. There can be no chance for an argument respecting the spaces broader at the grinding surfaces than at the necks of the teeth, for they cannot be closed up except in defiance of all laws of mechanical force. As touching the V spaces having their broadest opening at the lingual or palatine aspect, it would be impossibls to suppose, even if the disposition of the teeth was to turn on their longitudinal or perpendicular axis, that they would do so sufficiently to materially lessen the spaces between all the teeth, for in that event the teeth would eventually be contracted into the shape of a circle, and that of very small diameter. It is possible that certain individual teeth, or pairs of teeth—as, for instance, the bicuspids—might and sometimes would, incline toward one another, and by rotation lessen, or absolutely close up, the spaces between them; but, in that event, the spaces between each of these teeth and its mesial and distal neighbor would be increased. It is difficult to think it possible for such spaces as Dr. Arthur proposes to make between the molars and bicuspids, ever to become materially lessened by the change in position of the teeth; and I think we must dismiss this ground of objection to the practice, as not being sustained by reason or philosophy.

Another objection suggests itself to many who are mainly in favor of the general plan, and that is, that although the spaces may be ample at the broadest end of the two wedges, yet that the narrowing to a point must be so gradual that they fear the place for the lodgement of decomposing food, or other deleterious agents, would rather be increased than diminished by this proposed separation. It is certainly true that the spaces must be very small, as the sides of the separation approach one another, and though perhaps the teeth are actually touching at only a single point, they still are in so close proximity, unless separated to an extent which I

do not understand Dr. Arthur to advocate, as to *seem* in contact for a considerable extent of their surfaces. This would appear to be a possible objection, but experience may teach us that it is unsound. It clearly can have little weight, if the proper care is observed on the part of the patient with regard to cleansing the teeth; nor can I conceive that it should influence us at all, unless we are convinced that the teeth are more exposed to decay than they would be if not separated. And again, I think it would need to be shown that the teeth would decay much more readily when thus separated, in order to counterbalance the benefits which would be derived, of easier access to the surfaces, for the purposes of examination and cleansing.

Another objection, very strongly urged by many, is the great annoyance to the patient, and injury to the gums, from the crowding of food between the teeth, and its pressure upon the soft parts, which would probably result from this treatment. This objection can only be met by the teachings of experience. We need waste no time in argument upon a point which can only be determined by the actual demonstrations of long and various experiments; but it may not be amiss to say that it is very possible that those who urge this objection so strongly do so without fully and clearly understanding the character of the separations proposed, and may have in their minds the complaints they have heard from their patients where the separations were of a very different character.

Again, objection is made to the cutting away of so great an extent of masticating surface. I apprehend this objection cannot stand for a moment, if it is once conceded that Dr. Arthur's method will accomplish what he claims for it. In fact, let this once become established, and all objections melt before the inexorable logic of demonstrated success.

It now remains for us to examine what experience teaches regarding this method, and to inquire if that teaching and the promptings of reason indicate the propriety or otherwise of our pursuing this method.

In the line of the testimony of experience, we have that of Dr. Arthur himself, whose testimony is by far the most important of any others, because his experience extends over a much longer period of time. It may be urged by some that his evidence is not to be too implicitly trusted, because he is an interested party, and that he would naturally think that methods of his own devising were better than all others. This might, or might not, have weight with those who are acquainted with Dr. Arthur; but to those who do not know him, there are some points worthy of consideration in estimating the value of his evidence. He certainly presents a most striking peculiarity in contrast with the large majority of those in our profession who have, or have claimed to have, devised new methods of practice. While most of these have rushed before the profession with their new-born ideas barely crystalized into intelligible form and expression, and have announced their new methods to be pre-eminent over all others, and sure to stand, though unsupported by the test of any experience worthy of the name, he comes to us after, as he tells us, a careful practice of the method for a period of twenty-five years. He presents us this method as the result of very mature and careful thought and study, indorsed by his long experience of its success. He tells us that he did not rush into this practice in a headlong manner, but carefully following the indications of his convictions, step by step feeling his way, he was brought to the conclusions which have resulted in his "method for the prevention and treatment of the decay of the teeth." To me, and I think to most fair-minded individuals, the testimony of such a man, as to even his own methods, challenges attention and credence in no ordinary degree. His evidence is to the unqualified success of his treatment. We cannot ignore his testimony; it must have *great* weight, even if we are convinced that it should be received with some degree of caution. We have no other testimony covering the same ground, but perhaps some may be found tending to corroborate his, among those who have not pretended to follow his method as such. It has probably been the fortune of most of the older practitioners who, from fifteen to twenty years ago, were in the habit of separating the bicuspid and molar teeth, for the purpose of filling, to the extent, sometimes, of sacrificing one-half or two-thirds of their grinding surfaces. I think it cannot have escaped the observation of those who have seen many of this class of operations, that as compared with those equally well performed by others, or even by the same individuals, where the separations had been slight, or only temporary, or without the broad V shape, the first mentioned operations were after the lapse of years, standing evidence not only of the operator, but of the value of the space, as tending to the preservation of the teeth; while those of the other class, even if of much more recent date, would more frequently present the evidence of the necessity for their renewal, even if the teeth were not already so extensively destroyed as almost to preclude the possibility of saving them. I do not refer to isolated cases now, but to observations covering a large number, and where the opportunities for comparison have existed. It has been my fortune to have the opportunity to see a large number of operations, in a large number of mouths, made by the same individual, one of the first operators in the country. These operations have been of various dates, covering a period of eighteen years. The earlier of them were almost or entirely made with the very broad V shape referred to. Some of the later ones were of the other class, such as are denominated contour, or nearly so. The difference has been *very* marked, so much so as to challenge my very particular attention. Others have made the same observations regarding this same gentleman's operations, in other cases which have fallen under their observation, and not under mine. This would seem to furnish a very strong case in favor of the method of using the free opening—much stronger, of course, by reason of noticing the comparative results of the different classes of operations from the same hand, and that the hand of a master, than though the different classes were compared as performed each by a different operator.

Still, the comparison of the results of such different classes of operations, made each by different individuals, must furnish us, if uniform in result, and covering a long period of time, incontestible evidence of the superiority of one or the other method. My own observation, and I doubt not that of most of you, has confirmed the opinion that the broad V-shaped spaces are exceedingly valuable, as securing the greater permanence of operations and preventing further decay on the proximal surfaces of the molars and bicuspids. The separations to whieh I now refer, the results of which, having particularly attracted my notice, have been *very great*, as was mentioned, sometimes to the extent of cutting away one-half or more of the grinding surfaces of bicuspids, and even the same of molars. Now I do not understand Dr. Arthur to propose any such extensive separations, though his work is rather indefinite in its information respecting the width of the spaces. It, therefore, remains to be proven by demonstration whether the smaller spaces with the double V shape will prove as valuable as it seems to be established the larger singleV shape is for the purposes under consideration. If we accept the testimony of Dr. Arthur, and give due weight to such other corroborative testimony as observation and experience furnish regarding the effect of spaces between the teeth, I am constrained to think that there can be very little urged against his method that will bear the unprejudiced criticism of reason and philosophy. It is to be regretted that his book was not addressed more exclusively to the profession, and that it does not enter more fully into details of processes—as, for instance, to the breadth of space which should be secured in separating; and in many other particulars it might have been more explicit and more instructive. I had hoped to secure from him a few models to present to you, practically demonstrating the shapes and other characteristics of the spaces which he advocates but he could not furnish them to me, and so we must exercise our own judgment in the matter, and get at an understanding of his method to the best of our ability, unaided by such guides.

Now in view of what has been thus far advanced, may we not hope that a better way is opening before us?

I am not prepared to say that there can be no better way, or to dogmatically assert that such practice as Dr. Arthur proposes will be successful beyond the peradventure of a doubt, but I do think that I may safely ask your most thoughtful and unprejudiced consideration of this method, believing that it does at least lead in the right direction.

There is one point more only, in Dr. Arthur's book, to which I wish to allude, but it seems to me of so much importance as not to justify my passing it by. Page 154, he says:—"I have said that this method of treatment is simple; it is so. All the requisite operations are certainly within the capacity of dentists of very moderate ability."

Here I should take issue with him most decidedly. His language here seems to imply that very little skill is requisite for such operations, an inference which would be likely to lead to most disastrous results, and work serious injury by reason of hurried, slipshod, or insufficient operations of this character. In fact his own language immediately following the above quotation, seems to contradict this inevitable inference, for he says:—"But in order to be effective, this treatment demands the exercise of the nicest care, patient labor, and subsequent watchfulness of the most rigid character. No slovenly or hurried attention will suffice. All successful treatment of the teeth indeed, no matter what plan may be pursued, requires this exercise of care and attention in a much greater degree than is common."

Now my own experience has been that these operations are by no means so easy of execution as his remarks generally throughout the book, would lead one to suppose. On the contrary, I believe that for their most successful accomplishment they require a very high degree of skill. In fact, I have come to think that even the filling of so-called simplest cavities in the teeth, cannot be performed properly without more than a low order of capacity. I think Dr. Arthur has made the mistake that men are apt to make, when writing of practices and methods of which they are master, that is of seeming to underrate the importance of the operation, or the extent of their own ability

Things that are easy of accomplishment to such men may be very difficult to compass for the majority, and one is apt to think and speak of things which are so familiar to himself, as though they would of necessity be just as apparent, or easy of accomplishment to all.

Such an error should be carefully guarded against, and no one should imagine for a moment that the teeth can be successfully treated, without a more than ordinary degree of skill, or by the employment of any but their best and highest endeavors.

We have now considered as fully as such a paper as this admits, the only proposed plan offered as yet, as superior to old methods for the prevention of decay. The treatment of it, after it has occurred, need not occupy our attention for more than a brief reference, as the only peculiar feature which would characterize it, under the method which has been discussed, would be that of the separation to be made, practically the same as those for the prevention.

Of course, after decay has progressed beyond a certain point, its removal by cutting becomes impracticable, and it then only remains for us to fill thoroughly upon the surfaces presenting after the separations are made, in accordance with the foregoing method. I cannot close this already too lengthy paper, without saying that the thanks of the profession are certainly due to Dr. Arthur for thus placing before them a method thoroughly systematiezd (for whether perfect or not, it cannot be all wrong,) as it is the first method having this characteristic, ever presented to us. It is possible that the time will come when we shall acknowledge our gratitude to him for having directed us in a better way, and when our patients, too, will bear his name in grateful remembrance. I think it probable that time will come. Finally, let me urge you again, carefully to examine and test this method, and not to condemn it by reason of strongly fixed prejudices in favor of other and probaly more popular ones. Let its merits alone influence your decision, and if, after an impartial trial, it fails in your judgment to bring the desired results, rest not in your endeavors to attain to a better practice than any now known to us.

In such pursuit alone can we fill the proper measure of our responsibility.

